# Functional Insights Into the Role of *gppA* in (p)ppGpp Metabolism of *Vibrio cholerae*

**DOI:** 10.3389/fmicb.2020.564644

**Published:** 2020-09-29

**Authors:** Dipayan Rakshit, Shreya Dasgupta, Bhabatosh Das, Rupak K. Bhadra

**Affiliations:** ^1^Infectious Diseases and Immunology Division, CSIR-Indian Institute of Chemical Biology, Kolkata, India; ^2^Molecular Genetics Laboratory, Infection and Immunology Division, Translational Health Science and Technology Institute, NCR Biotech Science Cluster, Haryana, India

**Keywords:** *Vibrio cholerae*, stringent response, (p)ppGpp, GppA, Ppx, amino acid starvation, glucose starvation

## Abstract

The stringent response, an adaptive response to nutrient limitation and exposure to xenobiotics in bacteria, is mediated by two intracellular signaling molecules, pppGpp and ppGpp, together called (p)ppGpp. The cellular level of (p)ppGpp in bacterial cells is controlled by the Rel/Spo family of proteins. In the cholera pathogen, *Vibrio cholerae*, (p)ppGpp metabolism is regulated by the products of at least three genes *relA*, *spoT*, and *relV*. In this study, we identify and characterize the function of the guanosine-5′-triphosphate 3′-diphosphate pyrophosphatase A (GppA) encoding gene *gppA* of *V. cholerae*. Genomic analysis indicates that the *gppA* locus is conserved in vibrios and organized as a bicistronic operon along with the *rhlB* gene. We engineered the genome of *V. cholerae* to develop different mutants devoid of GppA and/or other phosphate metabolic enzymes. Our findings indicate that in *V. cholerae*, GppA plays an important role in the conversion of pppGpp to ppGpp during amino acid deprivation but not during glucose starvation. Quantitative analyses of the *gppA* transcript level reveal its differential expression pattern at different growth phases and starvation conditions. It has been observed that the GppA deficiency during amino acid starvation condition could be complemented by overexpressing the exopolyphosphatase coding gene *ppx* of *V. cholerae*. By deletion analysis, we further demonstrate that the amino and carboxy terminal sequences flanking the Ppx-GppA motif of the GppA protein of *V. cholerae* are also important for its enzymatic function.

## Introduction

**FIGURE 1:**
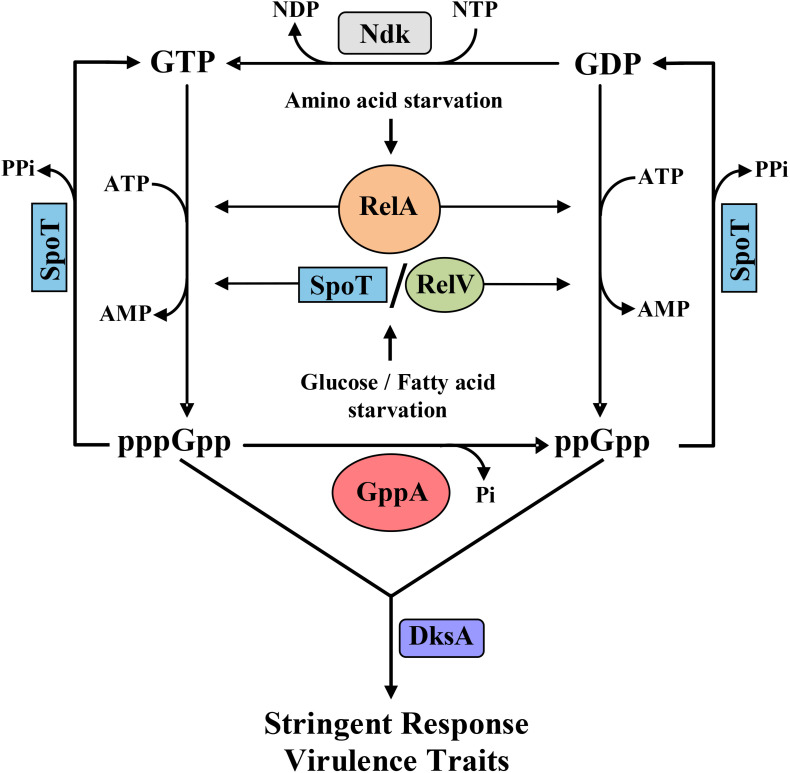
Schematic diagram showing the stringent response (SR) metabolic cycle involving different enzymes of *V. cholerae*. As shown, the RelA enzyme is activated during amino acid starvation, while glucose or fatty acid starvation induces SpoT and RelV followed by synthesis of pppGpp and ppGpp using guanosine triphosphate (GTP) and guanosine diphosphate (GDP) as substrates, respectively. pppGpp is converted to ppGpp by the enzyme GppA (this study). SpoT, the bifunctional enzyme, has a strong hydrolase activity and thus hydrolyzes pppGpp and ppGpp to GTP and GDP, respectively. GDP is subsequently converted to GTP by the nucleotide diphosphate kinase (Ndk) enzyme. Synthesized pppGpp/ppGpp molecules with the help of the regulatory protein DksA controls the SR and several critical virulence traits of *V. cholerae* like cholera toxin production, biofilm formation, motility, and protease expression.

The enteric pathogen *Vibrio cholerae* faces various physicochemical stresses while living within or outside of the human intestine. Among multiple environmental stresses, nutritional stress is important for survival since it determines the growth and multiplication of the pathogen under such conditions. Like other bacterial pathogens, *V. cholerae* has evolved with complex gene regulatory networks to cope with various environmental stresses, among which the stringent response (SR) is important. The SR is typically characterized by strong repression of transcription of stable RNAs like transfer RNAs (tRNAs) and ribosomal RNAs (rRNAs), etc. (Cashel et al., 1996; Potrykus and Cashel, 2008; Gaca et al., 2015), upregulation of transcription of genes coding for the enzymes involved in amino acid biosynthesis (Stephens et al., 1975; Choy, 2000), and inhibition of replication (Wang et al., 2007). Regulations of all the above stated cellular processes under nutritional stress conditions are crucial for the survival of bacteria. The SR is essentially managed by two intracellular small signaling molecules, guanosine 3’-diphosphate 5’-triphosphate (pppGpp) and guanosine 3’,5’-bis(diphosphate) (ppGpp), together called (p)ppGpp (Cashel et al., 1996; Potrykus and Cashel, 2008). While in *Escherichia coli*, intracellular (p)ppGpp metabolism is controlled by two multidomain containing proteins RelA and SpoT, in *V. cholerae*, apart from these two enzymes, a small alarmone synthetase, called RelV, is also involved in (p)ppGpp metabolism as shown in Figure 1 (Haralalka et al., 2003; Das and Bhadra, 2008; Das et al., 2009; Dasgupta et al., 2014). RelA, the product of the *relA* gene, is a ribosome-associated protein and responsible for (p)ppGpp synthesis under amino acid starvation (Cashel et al., 1996). RelA is able to synthesize pppGpp and ppGpp by using guanosine triphosphate (GTP) and guanosine diphosphate (GDP) as substrates, respectively (Mechold et al., 2002; Sajish et al., 2009; Kudrin et al., 2018). On the other hand, SpoT, the product of the *spoT* gene, has a strong (p)ppGpp hydrolase activity and weak (p)ppGpp synthetase activity under different stress conditions (Xiao et al., 1991; Seyfzadeh et al., 1993; Das et al., 2009). Interestingly, in *V. cholerae*, the RelV enzyme, which is unique among Gram-negative bacteria, also contributes in (p)ppGpp synthesis under glucose and fatty acid starvations (Das et al., 2009; Dasgupta et al., 2014). Furthermore, unlike RelA and SpoT, RelV is a small single-domain-containing protein (Das et al., 2009; Dasgupta et al., 2014). In *E. coli*, apart from RelA and SpoT, another well-characterized enzyme, called guanosine pentaphosphate 5′-phosphohydrolase A (GppA), is associated with the (p)ppGpp metabolic cycle (Somerville and Ahmed, 1979; Hara and Sy, 1983; Keasling et al., 1993; Mechold et al., 2013). The main function of GppA is to convert pppGpp to ppGpp by removing the terminal γ-phosphate from 5’-position of pppGpp. Initial mutational analysis by Somerville and Ahmed (1979) indicated the presence of the GppA enzyme in *E. coli* for the conversion of pppGpp to ppGpp. However, Hara and Sy (1983) were the first to purify, characterize, and show the substrate specificity of GppA enzyme of *E. coli*. Recently, Mechold et al. (2013) have successfully manipulated the expression of GppA to demonstrate differential accumulation of ppGpp and pppGpp in *E. coli* cells, which allowed them to conclude that pppGpp is less potent than ppGpp with respect to regulation of growth rate, RNA/DNA ratios, ribosomal RNA P1 promoter transcription inhibition, threonine operon promoter activation, RpoS induction, etc. After the discovery of GppA, another phosphatase, called exopolyphosphatase (Ppx), was identified in *E. coli* (Akiyama et al., 1993). Ppx has been shown to hydrolyze the inorganic polyphosphate (polyP) to P_*i*_ (Akiyama et al., 1993). PolyP, a polymer of hundreds of phosphate residues, accumulates in *E. coli* and in other microbes in response to various stresses, including nutritional starvation (Kuroda et al., 2001). PolyP in *E. coli* and in other bacteria is synthesized by the enzyme polyphosphate kinase (Ppk), and the genes encoding Ppk and Ppx are usually physically linked (Akiyama et al., 1993; Kuroda et al., 1997, 2001; Ogawa et al., 2000). Extensive *in silico* analysis further indicated that GppA and Ppx are homologous proteins, and both of them belong to the sugar kinase/actin/hsp70 superfamily (Reizer et al., 1993; Koonin, 1994). It has been shown that like Ppx, the GppA enzyme of *E. coli* also has exopolyphosphatase activity, and thus, it is a bifunctional enzyme (Keasling et al., 1993). Similarly, a modest pppGpp hydrolase activity of *E. coli* Ppx has previously been demonstrated (Kuroda et al., 1997). It may be mentioned here that the members of Actinobacteria, like mycobacteria, corneybacteria, actinomycetes, etc., as well as certain other Gram-negative pathogens may encode homologous “Ppx-GppA” motif containing enzymes instead of separate Ppx and GppA proteins. The “Ppx-GppA” family of proteins share homology with both Ppx and GppA, and thus, they may have the ability to hydrolyze both polyP and pppGpp as substrates (Kristensen et al., 2004, 2008; Choi et al., 2012; Malde et al., 2014; Song et al., 2019).

Evolution of GppA supports that ppGpp is most likely the crucial molecule for the execution of SR in bacteria. Over the years, our group has characterized and established the entire SR regulatory genetic circuits including the discovery of the unique (p)ppGpp synthetase gene *relV* in *V. cholerae* as shown in Figure 1 (Haralalka et al., 2003; Das and Bhadra, 2008; Das et al., 2009; Pal et al., 2011, 2012; Dasgupta et al., 2014). In this (p)ppGpp metabolic cycle, conversion of GDP to GTP is carried out by the enzyme nucleoside diphosphate kinase, called Ndk, which has been characterized by the other workers (Dar et al., 2011). We have shown that the SR regulatory circuit is also linked with the modulation of certain critical virulence-related traits of *V. cholerae* (Haralalka et al., 2003; Pal et al., 2012; Basu et al., 2017; Basu and Bhadra, 2019). However, at present, no information is available about the functional aspect of the *gppA* gene in the cholera pathogen, the product of which converts pppGpp to ppGpp. Furthermore, very little information is available about the level of pppGpp and ppGpp under amino acid or glucose starved condition. Like in *E. coli*, *V. cholerae* genome also carries the physically linked *ppk-ppx* genes, which are involved in polyP metabolism (Heidelberg et al., 2000; Ogawa et al., 2000). In this report, we have carried out extensive genetic analysis of the *gppA* gene and show that the product of the gene is linked with the (p)ppGpp metabolism in *V. cholerae* (Figure 1). Like in *E. coli*, Ppx is also homologous to GppA in *V. cholerae*; therefore, mutational approaches were adopted to analyze the function of *ppx* in the presence or absence of the *gppA* gene. It was found that the overexpression of *V. cholerae* Ppx can complement the GppA function.

(Part of this work has been presented in the 54th United States–Japan Joint Panel Conference on Cholera and Other Bacterial Enteric Diseases held on 10–13 December 2019 at Osaka University, Osaka, Japan).

## Materials and Methods

### Bacterial Strains, Plasmids, Media, and Growth Conditions

Bacterial strains and plasmids used in this study are described in Table 1. For cloning purpose, the plasmid pBluescript II KS(+) and *E. coli* DH5α strain were used unless stated otherwise. Both *E. coli* and *V. cholerae* cells were routinely grown in Luria broth (LB; Difco, United States) at 37°C with aeration, and for plate culture, Luria agar (LA; Difco) was used, which contained 1.5% (w/v) Bacto agar (Difco) as described earlier (Haralalka et al., 2003). Antibiotics (Sigma-Aldrich, United States) were used in the following concentrations: ampicillin (Ap), 100 μg ml^–1^; kanamycin (Km), 40 μg ml^–1^; streptomycin (Sm), 100 μg ml^–1^; chloramphenicol (Cm), 3 μg ml^–1^ for *V. cholerae*; and 30 μg ml^–1^ for *E. coli*. Bacterial strains were stored at −70°C in LB containing 20% (v/v) sterile glycerol (Verma et al., 2019) and were always taken out before doing experiments to avoid the development of any uncharacterized mutation. The growth of bacterial cultures was monitored by measuring optical density at 600 nm (OD_600_) using a spectrophotometer (Hitachi, Model U-5100).

**TABLE 1:** Bacterial strains and plasmids used in this study.

*Strains*	Relevant genotype and/or phenotype	Source/reference
***V. cholerae***		
N16961	Wild type, O1 serogroup, biotype El Tor, Sm^*r*^	Lab stock
NΔgAK (Δ*gppA*)	N16961 Δ*gppA*::*kan*; Km^*r*^Sm^*r*^	This study
NΔPPX (Δ*ppx*)	N16961 Δ*ppx*::*cam*; Cm^*r*^Sm^*r*^	This study
NΔgPx (Δ*gppA*Δ*ppx*)	NΔgAK Δ*ppx*::*cam*; Cm^*r*^Km^*r*^Sm^*r*^	This study
***E. coli***		
DH5α	F’ *endA1 hsdR17 supE44 thi-1 recA1 gyrA96 relA1*Δ(*argF-lacZYA*) *U169* (Φ80d*lacZ*ΔM15)	Lab stock
SM10λ*pir*	*thi thr leu tonA lacY supE recA*::RP4-2-Tc::Mu λ*pir R6K*	Lab stock
**Plasmids**		
pBluescript II KS(+)	ColE1, high-copy-number cloning vector; Ap^*r*^	Stratagene
pKAS32	*rpsL* suicide vector with *ori*R6K *mob*RP4; Ap^*r*^	Lab stock
pBAD24	pBR322 origin, L-arabinose inducible vector; Ap^*r*^	Lab stock
pROTet.E	ColE1 origin, high copy number expression vector; Cm^*r*^	BD Biosciences
pUC4K	Source of *kan* resistance gene cassette; Ap^*r*^ Km^*r*^	Pharmacia
pKΔgAK	2.0-kb Δ*gppA::kan* allele cloned in *Kpn*I/*Sac*I sites of pKAS32; Ap^*r*^ Km^*r*^	This study
pKPXUDCam	1.7-kb Δ*ppx::cam* allele cloned in *Kpn*I/*Sac*I sites of pKAS32; Ap^*r*^ Cm^*r*^	This study
pBADcam	0.8-kb *cam* resistance gene cassette from *Eco*RV digested pROTet.E was cloned in *Sca*I site of pBAD24; Cm^*r*^	This study
pGppA	1.6-kb *gppA* ORF of N16961 cloned in *Eco*RI*/Pst*I sites of pBADcam; Cm^*r*^	This study
pPpx	1.6-kb *ppx* ORF of N16961 cloned in *Xba*I/*Hin*dIII sites of pBADcam; Cm^*r*^	This study
pGppA1-272	pGppA with 678-bp deletion from 3′-end of *gppA* ORF; Cm^*r*^	This study
pGppA1-303	pGppA with 585-bp deletion from 3′-end of *gppA* ORF; Cm^*r*^	This study
pGppA1-308	pGppA with 570-bp deletion from 3′-end of *gppA* ORF; Cm^*r*^	This study
pGppA1-310	pGppA with 564-bp deletion from 3′-end of *gppA* ORF; Cm^*r*^	This study
pGppA1-313	pGppA with 555-bp deletion from 3′-end of *gppA* ORF; Cm^*r*^	This study
pGppA13-497	pGppA with 36-bp deletion from 5′-end of *gppA* ORF; Cm^*r*^	This study
pGppA18-497	pGppA with 51-bp deletion from 5′-end of *gppA* ORF; Cm^*r*^	This study
pGppA20-497	pGppA with 57-bp deletion from 5′-end of *gppA* ORF; Cm^*r*^	This study
pGppA23-497	pGppA with 66-bp deletion from 5′-end of *gppA* ORF; Cm^*r*^	This study
pGppA18-310	pGppA with 51-bp deletion from 5′-end and 564-bp deletion from 3′-end of *gppA* ORF; Cm^*r*^	This study

*Ap, ampicillin; Cm, chloramphenicol; Km, kanamycin; Sm, streptomycin.*

### Molecular Biological Methods

For chromosomal and plasmid DNA preparations, restriction enzyme digestion, DNA ligation, bacterial transformation, conjugation, agarose gel electrophoresis, etc., standard molecular biological methods (Ausubel et al., 1989) were followed. Restriction and nucleic-acid-modifying enzymes were purchased from New England Biolabs, Inc., United States and were used essentially as instructed by the manufacturer. Electrocompetent *V. cholerae* cells were made as described previously from this laboratory (Das and Bhadra, 2008), and transformants were selected on LA plates containing appropriate antibiotics.

### Construction of Plasmids and Mutants

Throughout the study, the expression vector pBADcam (Table 1), a derivative of pBAD24 (∼4.5 kb; Ap^*r*^; Table 1) carrying the Cm gene (*cam*) cassette, was used. To construct pBADcam, the plasmid pPROTet.E (2.2 kb; Cm^*r*^; Table 1) was digested with *Eco*RV, and ∼0.8 kb digested product carrying the *cam* cassette was ligated to *Sca*I-digested pBAD24; the ligation mixture was transformed into *E. coli* DH5α. Among multiple clones obtained, one was selected for use and named pBADcam (∼5.3 kb; Cm^*r*^; Table 1). Details of the primers used in this study are described in Supplementary Table S1. The recombinant plasmid pGppA (Table 1) was constructed by amplifying the *gppA* Open Reading Frame (ORF) (∼1.6 kb) using the primers gppAorf*-*F/gppAorf*-*R (Supplementary Table S1) and the genomic DNA of *V. cholerae* O1 El Tor strain N16961 [Table 1; henceforth will be called wild type (Wt)], followed by double digestion with the restriction enzymes *Eco*RI*/Pst*I and cloning in similarly digested expression vector pBADcam. Similarly, the *ppx* ORF (∼1.6 kb) carrying plasmid pPpx (Table 1) was constructed using the primers ppxORF-F/ppxORF-R (Supplementary Table S1) and genomic DNA of *V. cholerae* Wt strain. The PCR amplified *ppx* ORF containing fragment was double digested with *Xba*I/*Hin*dIII and cloned in similarly digested vector pBADcam. For the construction of deletion mutants, we always used the suicide vector pKAS32 (Ap^*r*^; Table 1), which was maintained in *E. coli* SM10λ*pir* (Km^*r*^; Table 1). Details of the recombinant suicide plasmids, pKΔgAK and pKPXUDCam, constructed in this study are given in Table 1. Apart from *cam* gene, the *kan* gene cassette from the plasmid pUC4K (Table 1) was used for creation of a deletion allele of a desired gene of *V. cholerae*. To construct any deletion mutant of *V. cholerae*, the corresponding recombinant suicide plasmid carrying the mutant allele was conjugally transferred from *E. coli* SM10λ*pir* to *V. cholerae* cells, and the desired deletion mutant was selected by double crossover event using appropriate antibiotics as described previously (Haralalka et al., 2003; Das et al., 2009). Details of the deletion mutants of *V. cholerae* constructed in this study are given in Table 1.

For construction of plasmids carrying amino (N)- and/or carboxy (C)-terminal coding region deleted *gppA* gene, each of such desired fragments was PCR amplified using specific set of primers (Supplementary Table S1) and genomic DNA of *V. cholerae* Wt strain as templates followed by digestion of the desired fragment with *Eco*RI/*Pst*I and cloning of that DNA fragment in similarly digested vector DNA pBADcam. For amplification of 5′-end deleted fragment of *gppA* ORF each of the forward primers (Supplementary Table S1) always carried an artificially inserted ATG start codon. Similarly, for the amplification of 3′-end deleted fragment of the *gppA* ORF, each of the reverse primers (Supplementary Table S1) always carried an artificially inserted TAA stop codon. Restriction digestion and DNA sequencing further confirmed the recombinant plasmids.

### Detection of Intracellular (p)ppGpp Accumulation

Intracellular accumulation of (p)ppGpp in *V. cholerae* cells under various starvation conditions was determined by radiolabeling of bacterial cells with [^32^P]-H_3_PO_4_ (BRIT, Mumbai, India) using 3-(N-morpholino) propanesulfonic acid (MOPS) medium with different supplements followed by lysis of cells and thin layer chromatography (TLC) essentially as described previously (Haralalka et al., 2003; Das and Bhadra, 2008; Das et al., 2009). For (p)ppGpp labeling under no starvation conditions, the same MOPS medium mentioned above was used, but it contained all amino acids plus glucose. Bacterial cells were labeled as mentioned earlier (Haralalka et al., 2003; Das and Bhadra, 2008; Das et al., 2009) followed by autoradiography. Densitometric analysis of (p)ppGpp spots on autoradiogram was done using the ImageJ software^1^. Abundance of pppGpp in each lane relative to the total (pppGpp + ppGpp) was calculated by dividing the spot intensity of pppGpp with that of total intensity value of pppGpp + ppGpp.

### Reverse Transcriptase PCR Assay

For reverse transcriptase PCR (RT-PCR) assay, *V. cholerae* cells were grown in LB at an OD_600_ value of ∼1.0 followed by extraction of total cellular RNA using TRI Reagent (Sigma-Aldrich, United States) essentially as described previously (Pal et al., 2012; Basu et al., 2017; Basu and Bhadra, 2019). Purity and quantitation of prepared RNA were done spectrophotometrically. RT-PCR assay was carried out using the Qiagen One Step RT-PCR kit as directed by the manufacturer (Qiagen, Germany). The PCR-amplified product was checked by agarose gel electrophoresis using appropriate molecular size markers. To confirm the absence of any contaminating DNA in prepared RNA samples, PCR assay of each sample was also done with *Taq* DNA polymerase (Invitrogen). Lack of amplification in the absence of RT confirmed that the desired PCR product was generated only from complementary DNA (cDNA). For quantitative RT-PCR (qRT-PCR) assay, total cellular RNA was prepared from bacterial cells grown in LB medium to an OD_600_ value of ∼0.5, 1.0, or 1.5 as mentioned above. Reactions were done using the One Step SYBR^®^PrimeScript^TM^ RT-PCR Kit II essentially as described by the manufacturer (Takara Bio Inc., Japan). qRT-PCR assay was done using the CFX96^TM^ Real-Time System (Bio-Rad, United States). The primer set gppArtm-F/gppArtm-R (Supplementary Table S1) was used for qRT-PCR analysis. Relative expression values (*R*) were calculated using the equation *R* = 2^–(Δ^ *^*C*^_*T*_*^*target*^^–^ *^*C*^_*T*_*^*reference)*^, where *C*_*T*_ is the fractional threshold cycle. In each experiment, as an internal control, the *recA*-specific primers recA-F/recA-R (Supplementary Table S1) were used. Each assay was repeated at least thrice. To check the expression status of *gppA* during amino acid starvation, glucose starvation, and in the absence of any starvation, total cellular RNA was extracted from *V. cholerae* following the same experimental conditions as done for (p)ppGpp labeling experiment. The level of *gppA* transcripts was measured by qRT-PCR analysis. As an internal control, the *gyrA*-specific primers, gyrArtm-F/gyrArtm-R (Supplementary Table S1), were used as recommended earlier (He et al., 2012). As positive controls, we used the *V. cholerae* biofilm related regulatory genes, *vpsR* and *vpsT*, which have been shown to be upregulated during amino acid starvation (He et al., 2012). Each RT-PCR experiment was repeated at least thrice.

### DNA Sequencing and Computational Analyses

For confirmation of plasmid constructs, DNA sequencing was done using the BigDye^®^ Terminator v3.1 Cycle Sequencing Kit (Applied Biosystems Inc., United States) essentially as instructed by the manufacturer. The samples were run on an ABI3130 Genetic Analyzer using the pop-7 polymer (Applied Biosystems Inc.). DNA sequence data were compiled and analyzed by using the Chromas 1.45^2^. DNA sequences were obtained from J. Craig Venter Institute (JCVI)^3^, and protein domain information was obtained from Kyoto Encyclopedia of Genes and Genomes (KEGG)^4^. BLASTN and BLASTP programs were used to search for homologous nucleotide or protein sequences, respectively, in the database^5^. Clustal Omega software^6^ was used for the alignment of protein sequences and further modified through GeneDoc software version 2.7.000^7^. For designing primers, Primer3 software^8^ was used. For designing qRT-PCR primers, Primer Express 3.0 software (Applied Biosystems, United States) was used.

### Statistical Analysis

Where needed, pairwise comparison of data for each sample was analyzed for statistical significance using Student’s *t*-test.

## Results

### *In silico* Analysis of the *gppA* Gene of *V. cholerae*

**FIGURE 2:**
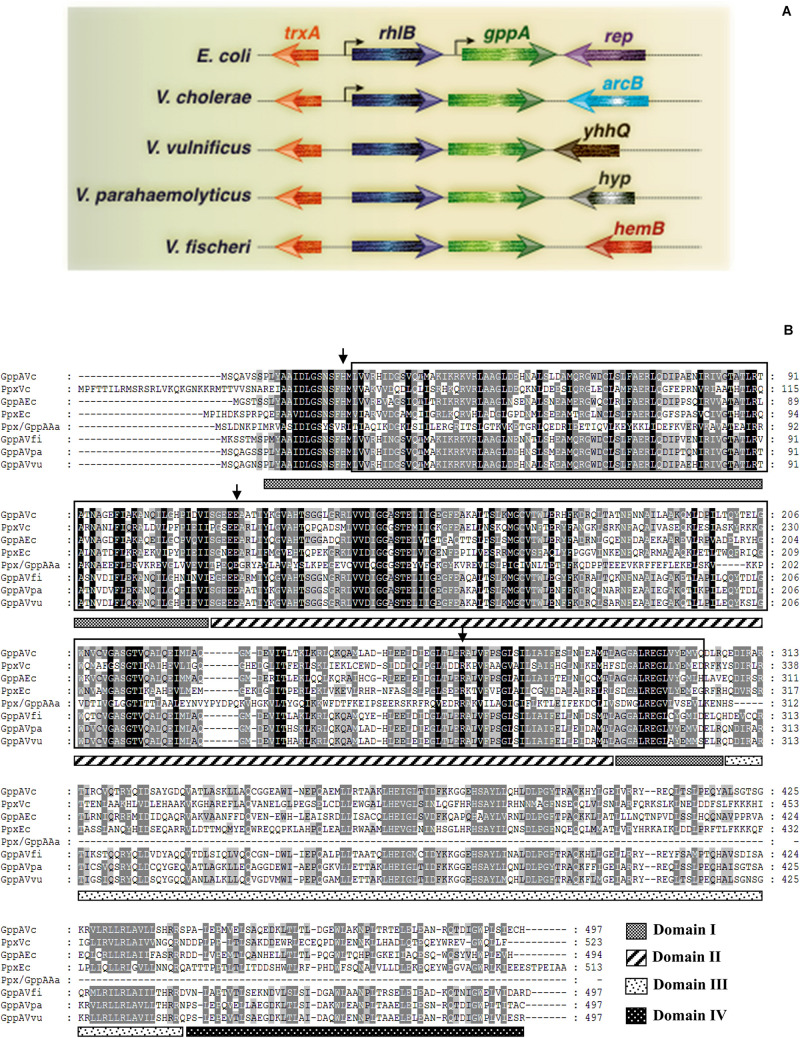
**(A)** Schematic diagram (not drawn to scale) showing genomic arrangement of the *gppA* gene in *V. cholerae*, *E. coli*, and other *Vibrio* spp. Names of different organisms are given in the left margin. Physically linked genes of *gppA* and their homologs are as indicated. Arrowheads indicate direction of transcription of genes. Thin gray line represents intergenic or chromosomal DNA. Bend arrows indicate putative monocistronic and bicistronic transcription of the *gppA-rhlB* genes in *E. coli* and *V. cholerae*, respectively. **(B)** Comparison of the amino acid sequences of Ppx-GppA motif containing proteins. Alignment was done using the Clustal Omega software (https://www.ebi.ac.uk/Tools/msa/clustalo/) and further modification by GeneDoc software version 2.7.000. Aligned proteins include GppA (GppAVc) and Ppx (PpxVc) of *V. cholerae* with those of *E. coli* GppA (GppAEc), *E. coli* Ppx (PpxEc), *A. aeolicus* Ppx/GppA (Ppx/GppAAa), *V. fischeri* GppA (GppAVfi), *V. parahaemolyticus* GppA (GppAVpa), and *V. vulnificus* GppA (GppAVvu). Dark and light shades indicate identical and similar amino acid, respectively. Boxed amino acid sequence denotes highly conserved Ppx-GppA motif (usually about 280 amino acids long). Small vertical arrows indicate conserved critical amino acid residues needed for phosphohydrolase activity. Four probable domains (domains I–IV) are indicated as different patterned bars. The domains are marked considering the PpxEc protein as a reference. Numbers in the right margin indicate amino acids length of each protein.

Since no information is currently available about the *gppA* gene of *V. cholerae*, BLASTN^9^ analysis of the whole-genome-sequenced *V. cholerae* Wt strain N16961 (Table 1) was carried out using the *E. coli gppA* gene sequence as a query. Such analysis indicated that the large chromosome of *V. cholerae* indeed carries a 1.5-kb long *gppA* gene (JCVI annotation no. VC0304). Comparison of the *gppA* gene sequence of *V. cholerae* indicated 63% nucleotide sequence identity with that of *E. coli* by using the BLAST Global Alignment Tool. While the *V. cholerae gppA* gene codes for a 497 amino acid long protein, the *E. coli* GppA is composed of 494 amino acids (see footnote 3). BLASTP analysis of *V. cholerae* GppA protein shows 57% identity (279 out of 491 amino acids) and 72% similarity with that of *E. coli* GppA. Genetic organization of the *gppA* gene in the genomes of *V. cholerae*, other *Vibrio* spp., and *E. coli* indicated that the *gppA* locus is highly conserved in vibrios (Figure 2A). BioCyc analysis^10^ suggested that the *gppA* gene along with its upstream region is physically linked with the *rhlB* gene (JCVI annotation no. VC0305), and they transcribe as a bicistronic operon. KEGG analysis of the 497 amino acid long GppA protein of *V. cholerae* revealed that it carries a highly conserved Ppx-GppA motif of 281 amino acids long (position 23 to 303), which is similar to the Ppx-GppA motif of *E. coli* GppA enzyme (Figure 2B). We also did BLASTN analysis for the *V. cholerae ppx* gene (JCVI annotation no. VC0722) and found that it encodes 523 amino acids long exopolyphosphatase. BLASTP analysis indicated that the *V. cholerae* Ppx has 40.5% identity with that of GppA (Figure 2B). Furthermore, amino acid sequence alignment of the GppA and Ppx proteins of *V. cholerae*, other *Vibrio* spp., *E. coli*, and thermophilic *Aquifex aeolicus* indicated substantial conservation of the residues including those needed for the pppGpp hydrolysis function (see Figure 2B). Analyses of the crystal structures of the *E. coli* Ppx and GppA proteins indicated the presence of four distinct domains, I–IV (Rangarajan et al., 2006; Song et al., 2019). It is evident that the *V. cholerae* Ppx and GppA proteins also carry similar domains as shown in Figure 2B.

### Transcriptional Organization of the *gppA* Operon of *V. cholerae*

**FIGURE 3:**
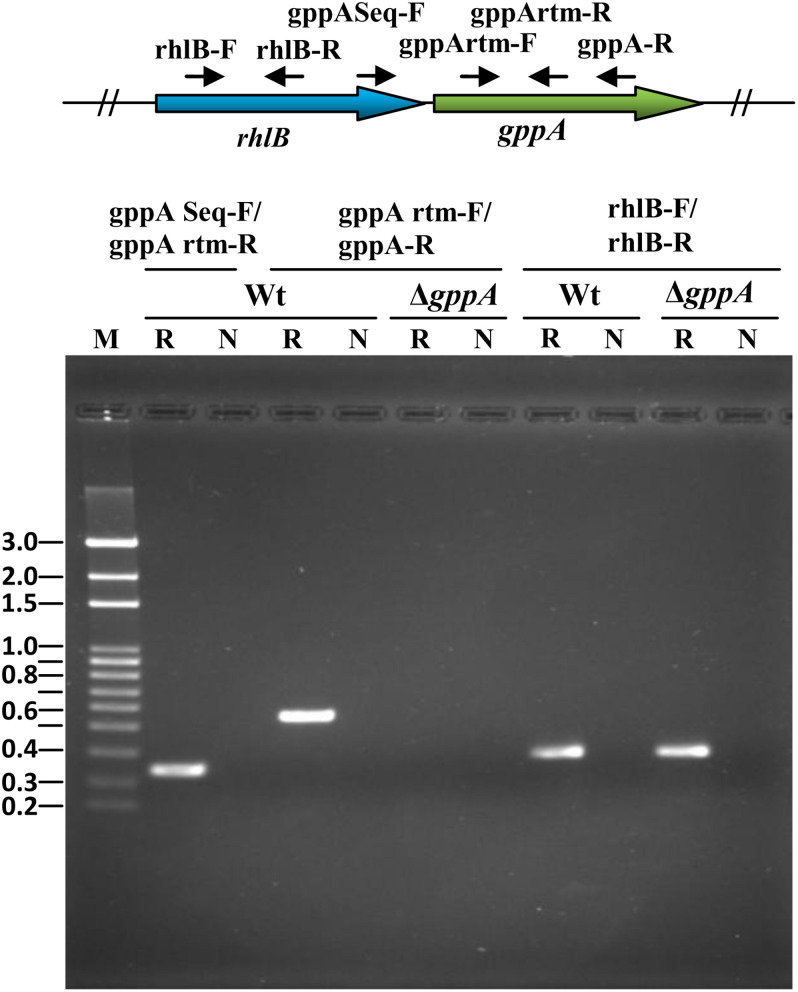
Reverse transcription PCR (RT-PCR) analysis to determine bicistronic arrangement of *V. cholerae rhlB*-*gppA* genes. Primers were designed exclusively from the *rhlB-gppA* locus, and small solid arrows indicate positions and directions of these primers. Primer sets used for RT-PCR analysis are indicated above each lane. Wt and Δ*gppA V. cholerae* strains are indicated above the lanes. “R” indicates RT with *Taq* polymerase enzyme, and “N” denotes use of only *Taq* DNA polymerase as a negative control. “M” denotes 100-bp DNA ladder used as molecular size markers, and their sizes (in kb) are given in the left margin.

Comparison of the *gppA* genetic loci of *V. cholerae* and *E. coli* revealed distinct difference in organization of different ORFs (Figure 2A). Bioinformatic analysis indicated that the *rhlB*-*gppA* genes of *V. cholerae* are arranged in a bicistronic operon since there is only a 7-bp gap between these two genes. The transcription orientations of the flanking ORFs of the *rhlB*-*gppA* operon are in reverse orientations (Figure 2A). In sharp contrast, the *E. coli gppA* gene appears to be organized as a monocistronic transcription unit since there is a 135-bp long intergenic region with predicted promoter sequence between the *rhlB* and *gppA* genes (Figure 2A). While the downstream region of *gppA* of *V. cholerae* contains the *arcB* gene encoding a sensor histidine kinase, in *E. coli*, the *rep* gene encoding an ATP-dependent DNA helicase is present in the same position (Figure 2A). In this study, we predicted that the *V. cholerae gppA* gene (JCVI annotation no. VC304) cotranscribes with the *rhlB* gene (JCVI annotation no. VC0305), and they are arranged in a bicistronic operon. To confirm this, *V. cholerae* cells were grown till late exponential phase (OD_600_ = 1.0), and total cellular RNAs were prepared for RT-PCR analysis. When the primer pair gppASeq-F (VC0305 ORF specific) and gppArtm-R (Supplementary Table S1) was used, as expected, a desired cDNA fragment of ∼0.33 kb in size was obtained (Figure 3). Similarly, when *gppA* or VC0305 ORF specific internal primers (gppArtm-F/gppA-R or rhlB-F/rhlB-R; Supplementary Table S1) were used, in each case, the desired cDNA fragment of size ∼0.56 or 0.37 kb, respectively, was obtained (Figure 3). The RT-PCR results confirmed that the *gppA* and VC0305 (*rhlB*) genes are indeed arranged in an operon, and they are cotranscribed. Next, we wanted to ensure that in the *gppA*-deleted *V. cholerae* strain NΔgAK (Table 1; henceforth will be called Δ*gppA*), the transcription of the neighboring gene *rhlB* (VC0305) was not affected. It is to be noted that during construction of the Δ*gppA* mutant, the closest *rhlB* ORF was kept intact; however, transcription of the *arcB* gene was not tested. For this purpose, the transcript status of *rhlB* in Δ*gppA* strain was examined by RT-PCR using the same set of primer rhlB-F/rhlB-R (Supplementary Table S1). While no cDNA was detectable for the *gppA* gene, *rhlB* ORF-specific primer set was indeed able to amplify the desired cDNA fragment of ∼0.37 kb using the Δ*gppA* mutant (Figure 3).

### GppA Modulates (p)ppGpp Homeostasis During Amino Acid Starvation

**FIGURE 4:**
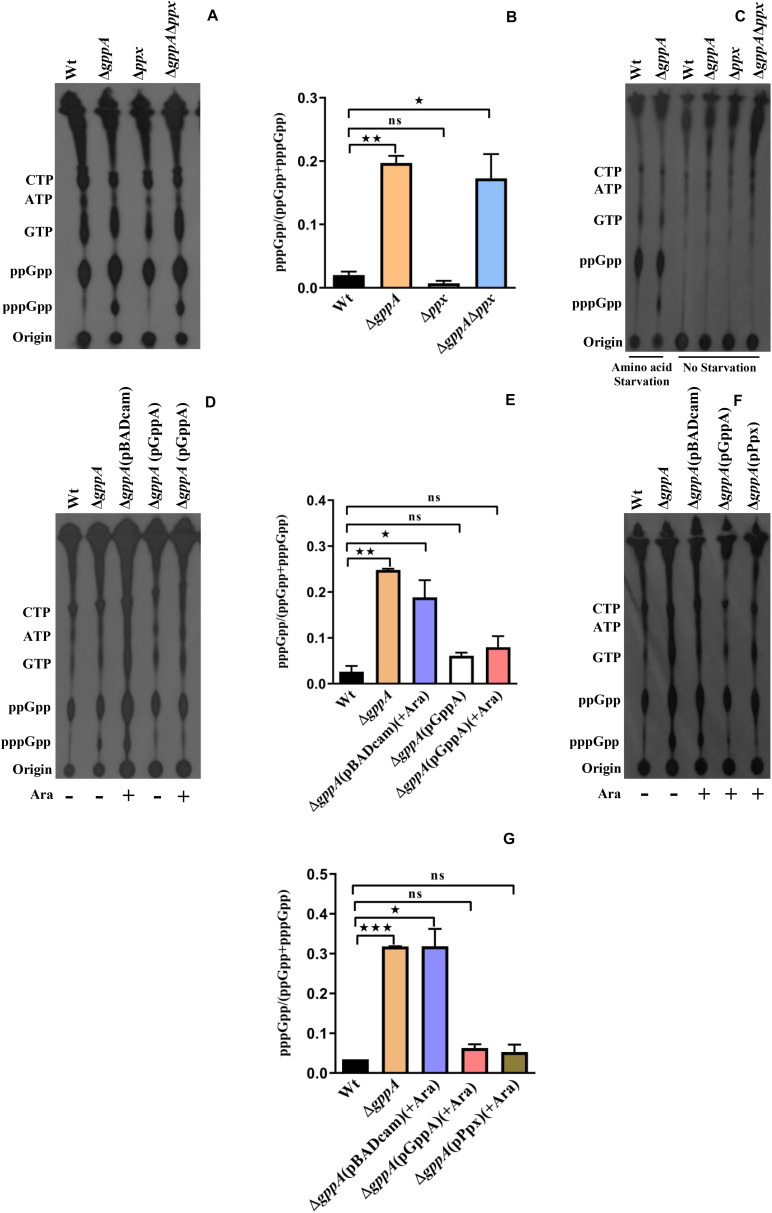
**(A)** Accumulation of (p)ppGpp in *V. cholerae* cells under amino-acid-starved condition. Bacterial cells were labeled with ^32^P-orthophosphoric acid, extracted, resolved by TLC followed by autoradiography. Strains used are Wt, *ΔgppA*, *Δppx*, and *ΔgppAΔppx*. Distinct accumulation of pppGpp in Δ*gppA* is evident. The presence of other labeled nucleotides including pppGpp/ppGpp is indicated in the left margin. **(B)** Densitometry of the (p)ppGpp spots detected in the autoradiogram was carried out by ImageJ software, and the ratio of pppGpp/(pppGpp + ppGpp) was plotted. Values represent the average of two independent experiments (*n* = 2), and one representative autoradiogram is shown in **(A)**. Error bars indicate standard deviation. (*P < 0.05; **P < 0.01; ns, non-significant). **(C)** Accumulation of (p)ppGpp in *V. cholerae* cells without any starvation (see details in text). Strains used are Wt, *ΔgppA*, *Δppx*, and *ΔgppAΔppx*. (p)ppGpp accumulation in Wt and Δ*gppA* under amino acid starvation condition served as controls. The presence of ^32^P-labeled nucleotides is indicated in the left margin. **(D)** Complementation analysis of the *ΔgppA* mutant of *V. cholerae*. Autoradiogram shows poor or no accumulation of pppGpp in *ΔgppA*(pGppA) strain in which *gppA* was expressed through the plasmid pGppA using 0.2% arabinose (Ara) as an inducer. Wt, Δ*gppA*, and Δ*gppA* carrying the empty plasmid [Δ*gppA*(pBADcam)] strains were used as controls. The presence of labeled nucleotides including pppGpp/ppGpp as indicated in the left margin. **(E)** Densitometric analysis of the (p)ppGpp spots was done as described in **(B)**. Values represent the average of two independent experiments (*n* = 2), and one representative autoradiogram is shown in **(D)**. Error bars indicate standard deviation. (*P < 0.05; **P < 0.01; ns, non-significant). **(F)** Autoradiogram showing (p)ppGpp accumulation in *ΔgppA* mutant by overexpressing *ppx.* Induction of both *gppA* and *ppx* expression through the plasmids pGppA and pPpx, respectively, was done using 0.2% arabinose as an inducer. Controls used are as given in **(D)**. The presence of labeled nucleotides including pppGpp/ppGpp as indicated in the left margin. **(G)** Densitometric analysis of the (p)ppGpp spots was done as described in **(B)**. Values represent the average of two independent experiments (*n* = 2), and one representative autoradiogram is shown in **(F)**. Error bars indicate standard deviation. (*P < 0.05; ***P < 0.001; ns, non-significant).

In order to understand the functions of GppA in regulating the intracellular pppGpp and ppGpp levels in *V. cholerae* under various starved conditions, intracellular (p)ppGpp labeling assay was performed using the Δ*gppA* mutant (Table 1) and its parental Wt strain. Before performing different experiments with the Δ*gppA* mutant, its growth kinetics in LB and M9 minimal medium were compared with that of Wt cells. However, in both the media, Δ*gppA* mutant showed no defect in growth when compared with the Wt (Supplementary Figures S1A,B). We have shown previously that the RelA of *V. cholerae* exclusively produces ppGpp under amino acid starved condition (Haralalka et al., 2003; Das and Bhadra, 2008). It may be argued here that under amino acid starvation, *V. cholerae* RelA may use GDP as a major substrate to synthesize ppGpp or it could use GTP as a substrate to produce pppGpp, which is rapidly converted to ppGpp by GppA. Interestingly, when Δ*gppA* cells were starved for amino acids along with its isogenic Wt strain and labeled with ^32^P followed by autoradiography, a significant increase (∼10-fold, see Figure 4) in the intracellular concentration of pppGpp in Δ*gppA* mutant was observed compared to the Wt (Figure 4A). The result suggests that, under amino acid starvation, GTP is also used as a substrate by the *V. cholerae* RelA to produce pppGpp followed by conversion to ppGpp by GppA. But in the case of Δ*gppA* mutant, which is devoid of GppA, this conversion is not possible, and thus, the mutant cells showed accumulation of substantial amount of pppGpp. This observation is also supported by densitometric analysis of the (p)ppGpp spots using the ImageJ software as shown in Figure 4B. This is further supported by the complementation assay using the Δ*gppA* cells carrying the *V. cholerae gppA* gene expressing plasmid pGppA (Table 1). As expected, such an assay and (p)ppGpp spot quantification clearly showed significant accumulation of ppGpp in Δ*gppA*(pGppA) cells like that of the parental Wt strain (Figures 4D,E). It is important to mention here that functional complementation by GppA was also observed in the case of Δ*gppA*(pGppA) in the absence of arabinose (Figure 4D), probably due to the leaky expression of *gppA* from the plasmid pGppA. By comparison of the profiles of pppGpp and ppGpp under amino acid starvation in *V. cholerae relA*^+^ *gppA*^+^ (Wt) and *relA*^+^ *gppA*^–^ (Δ*gppA*) genetic backgrounds, it appears that the GDP is a more preferred substrate for RelA than GTP (Figure 4A). In support of this conclusion, it may be mentioned here that the *E. coli* RelA is able to synthesize pppGpp and ppGpp using GTP and GDP as substrates, respectively (Sajish et al., 2009; Kudrin et al., 2018). It has been shown that pppGpp allosterically activates RelA leading to its increased efficiency in the synthesis of ppGpp from GDP (Kudrin et al., 2018). Thus, through all these genetic analyses, we first time established the function of GppA in *V. cholerae*.

### Overexpression of Ppx Complements Δ*gppA* Mutant During Amino Acid Starvation

Since the *ppx* gene of *V. cholerae* showed high homology with the *gppA* gene (Figure 2B), we also constructed *V. cholerae ppx* and *gppA ppx* deletion mutants to test the pppGpp level during amino acid starvation in the presence and absence of functional *ppx* gene. *V. cholerae ppx* and *gppA ppx* mutants constructed for this purpose were designated as NΔPPX (hereafter will be called Δ*ppx*) and NΔgPx (henceforth will be designated Δ*gppA*Δ*ppx*), respectively (Table 1). While Δ*ppx* behaved like Wt, the Δ*gppA*Δ*ppx* double mutant accumulated similar amount of pppGpp like in Δ*gppA* cells (Figure 4A). This was further verified by densitometric quantification of the (p)ppGpp spots (Figure 4B). To confirm that the spots shown in Figure 4A are indeed of (p)ppGpp, as a control experiment, the same set of bacterial strains was used to label (p)ppGpp using ^32^P without inducing any starvation. As expected, there was no accumulation of pppGpp and ppGpp in nutrient supplemented strains compared to the amino-acid-starved Wt and Δ*gppA* cells (Figure 4C). Apparently, the result suggests that the Ppx enzyme is probably unable to convert pppGpp to ppGpp, which could be due to its low expression/activity under the experimental condition used. To test this hypothesis, we have constructed a Ppx-overexpressing plasmid pPpx (Table 1). When Ppx was overexpressed in Δ*gppA* cells through the recombinant plasmid pPpx, it was able to complement the GppA function (Figure 4F). Densitometric analysis of the (p)ppGpp spots also supported this result as shown in Figure 4G. The result suggests that, under natural physiological condition, the basal level of Ppx probably has no or very weak pppGpp to ppGpp conversion activity. However, high abundance of the Ppx protein achieved by overexpression in Δ*gppA* cells most probably helped in overcoming this defect and converted pppGpp to ppGpp. The result warrants further studies to elucidate the exact role of Ppx in the metabolism of (p)ppGpp in *V. cholerae* under different nutritional stress conditions.

### GppA Mediated Conversion of pppGpp to ppGpp Is Not Observed During Glucose Starvation Condition

**FIGURE 5:**
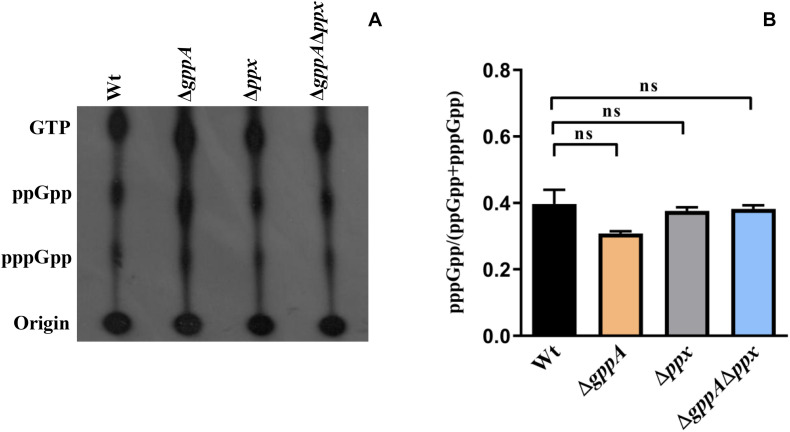
**(A)** Accumulation of (p)ppGpp in *V. cholerae* cells under glucose starvation. Strains used are: Wt, *ΔgppA*, *Δppx*, and *ΔgppAΔppx*. The presence of labeled nucleotides including pppGpp/ppGpp is indicated in the left margin. **(B)** Densitometry of the (p)ppGpp spots detected in the autoradiogram was carried out by ImageJ software and the ratio of pppGpp/(pppGpp + ppGpp) was plotted. Values represent the average of two independent experiments (*n* = 2) and one representative autoradiogram is shown in Panel A. Error bars indicate standard deviation. (ns = non-significant).

We further examined the status of pppGpp and ppGpp in *V. cholerae* Δg*ppA* mutant under glucose starvation. Interestingly, there was no difference in intracellular pppGpp and ppGpp levels between Δ*gppA* and isogenic Wt cells (Figure 5A). We extended our analysis to test the pppGpp and ppGpp levels in *V. cholerae* Δ*gppA*, Δ*ppx*, and Δ*ppx*Δ*gppA* mutants. As expected, no difference was observed in the intracellular pppGpp and ppGpp levels among Wt, Δ*gppA*, Δ*ppx*, and Δ*gppA*Δ*ppx* mutants either in the autoradiogram or by densitometric analysis (Figures 5A,B). At present, the mechanism(s) underscoring this contrasting function of *V. cholerae* GppA under amino-acid- and glucose-starved conditions is not clear. The possibility of the presence of interacting partner(s) that modulate GppA activity during glucose starvation or differential expression pattern of *gppA* during glucose and amino acid starvations cannot be ruled out and needs further investigation.

### Expression of the *gppA* Gene Increases During Stationary Growth Phase and Amino Acid Starvation Condition

**FIGURE 6:**
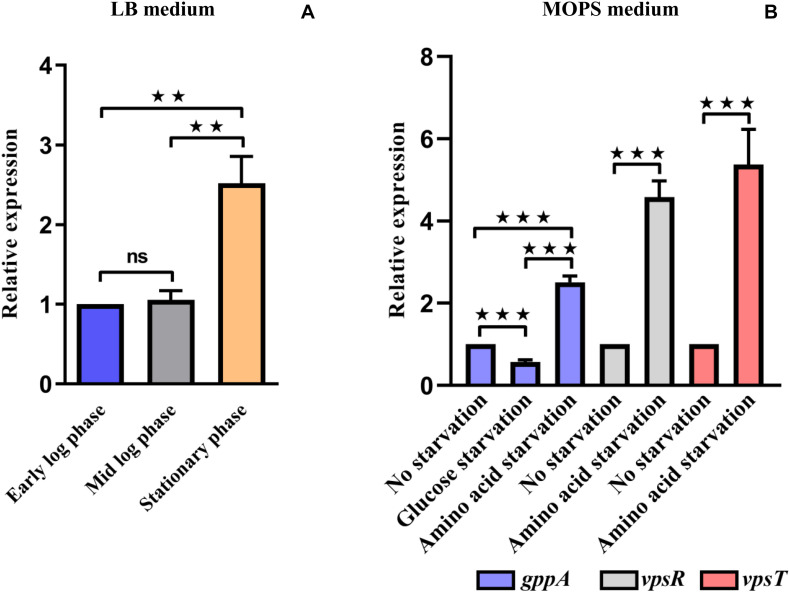
**(A)** Quantitative reverse transcription PCR (qRT-PCR) analysis of the expression of *V. cholerae gppA* gene in Luria broth (LB) medium under different growth phases as indicated (see details in text). Values represent the average of three independent experiments (*n* = 3). Error bars indicate standard deviation. (**P < 0.01; ns, non-significant). **(B)** qRT-PCR analysis of the expression of *V. cholerae gppA* gene under different starvation conditions in 3-(N-morpholino) propanesulfonic acid (MOPS) medium as indicated. As positive controls, the biofilm regulatory genes *vpsR* and *vpsT* were used (see details in text). Values represent the average of three independent experiments (*n* = 3). Error bars indicate standard deviation. (***P < 0.001).

After establishing the function of *gppA* gene in amino-acid-starved cells, we attempted to test its regulation, if any, during various growth phases and starvation conditions. To this end, we performed quantitative estimation of the *gppA* gene transcripts under various growth phases of *V. cholerae* cells in nutrient-rich LB medium using qRT-PCR assay. As shown inFigure 6A, there was significant increase (∼2.5-fold) in *gppA* transcripts level in stationary phase cells of *V. cholerae* Wt strain compared to that of early or mid-log phase cells. We further extended our analysis to explore the expression level of the *gppA* gene in *V. cholerae* under both amino acid as well as glucose-starved conditions with respect to non-starved condition in MOPS medium using qRT-PCR assay (see details in section “Materials and Methods”). Interestingly, qRT-PCR analysis indicated a significant downregulation (∼1.8-fold) of *gppA* expression in glucose-starved *V. cholerae* cells compared to non-starved bacteria (Figure 6B). On the other hand, it has been observed that *gppA* expression goes up (∼2.5-fold) during amino acid starvation compared to non-starved cells (Figure 6B). When we compared the *gppA* expression between glucose and amino acid starved cells, a significant (∼4.5-fold) upregulation was observed in the latter condition (Figure 6B). This differential expression pattern of *gppA* during amino acid and glucose starvations may be responsible for the lower level of pppGpp accumulation in Wt *V. cholerae* cells under amino acid starvation. To support this observation, as positive controls, we measured the expression levels of two biofilm regulatory genes *vpsR* and *vpsT* of *V. cholerae*. It has previously been reported that the *vpsR* and *vpsT* genes are upregulated during amino acid starvation conditions (He et al., 2012). Therefore, we compared the expression of *gppA*, *vpsT*, and *vpsR* in amino-acid-starved *V. cholerae* cells. As expected, the expression of both *vpsR* and *vpsT* genes are indeed upregulated (∼5-fold) during amino-acid-starved condition compared to non-starved *V. cholerae* cells (Figure 6B).

### Flanking Sequences Surrounding the Ppx-GppA Motif of GppA Are Important for Its Function

**FIGURE 7:**
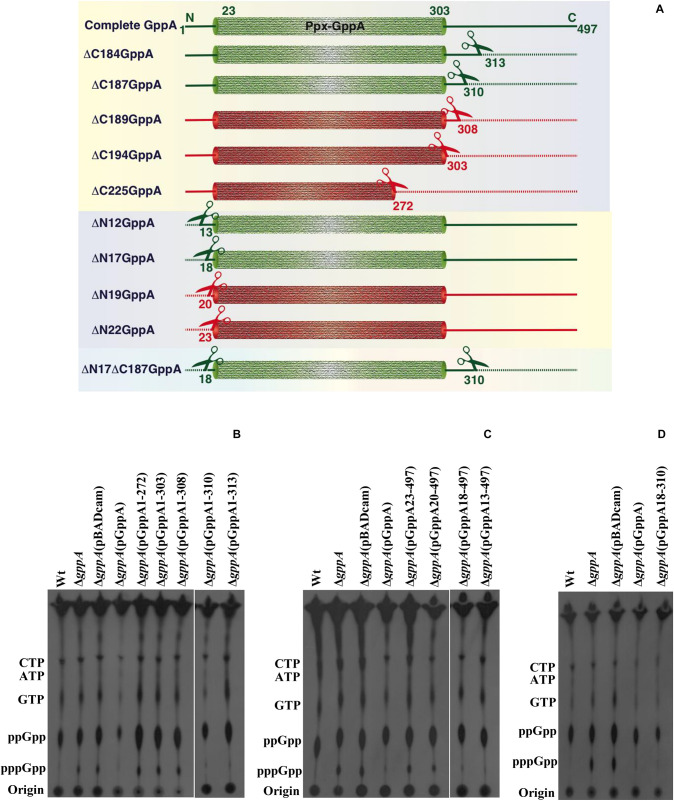
Functional analysis of the N- or/and C-terminal coding region deleted mutant alleles of the *gppA* gene of *V. cholerae.* **(A)** Schematic diagram (not drawn to scale) showing N- or/and C-terminal deleted regions of the *V. cholerae* GppA protein. Cylindrical-shaped portion of the GppA protein represents the putative Ppx-GppA motif, which spans from amino acid positions 23 to 303 as indicated. Scissors indicate position of each deletion, and dashed line represents deleted amino acid sequence from N- or/and C-terminal regions of GppA. Green and red colors denote functionally active and inactive GppA protein, respectively. N- and C-terminally deleted amino acid sequence of the GppA protein is symbolized by ΔN and ΔC, respectively, followed by the number of amino acids deleted as shown in the left margin. Intact GppA protein is 497 amino acids long, and the stretch of amino acid sequences present after deletion from N- or C-terminal of the GppA protein are shown at the corresponding ends. **(B)** Complementation analysis using C-terminal encoding sequence deleted alleles. Each C-terminally truncated GppA protein was expressed using 0.2% arabinose as an inducer in the Δ*gppA* mutant through the respective recombinant plasmid as shown above each lane. For the detection of pppGpp/ppGpp, thin layer chromatography (TLC) analysis of each strain was done followed by autoradiography. The presence of labeled nucleotides including pppGpp/ppGpp is indicated in the left margin. Autoradiogram shows complementation in Δ*gppA*(pGppA1-310) and Δ*gppA*(pGppA1-313) strains but not in Δ*gppA*(pGppA1-272), Δ*gppA*(pGppA1-303), and Δ*gppA*(pGppA1-308) strains. Wt, Δ*gppA*, Δ*gppA* carrying the empty vector pBADcam [Δ*gppA*(pBADcam)] and Δ*gppA* carrying the full-length GppA expressing clone pGppA [Δ*gppA*(pGppA)] were used as controls. **(C)** Complementation analysis using N-terminal encoding sequence deleted alleles. Each N-terminally truncated GppA protein was expressed using 0.2% arabinose as an inducer in the Δ*gppA* mutant through the respective recombinant plasmid as shown above each lane. TLC analysis of each strain was done followed by autoradiography. The presence of labeled nucleotides including pppGpp/ppGpp is indicated in the left margin. Autoradiogram shows complementation in strains Δ*gppA*(pGppA18-497) and Δ*gppA*(pGppA13-497) but not in strains Δ*gppA*(pGppA23-497) and Δ*gppA*(pGppA20-497). Control strains used as indicated in **(B)**. **(D)** Determination of minimal functional region of the GppA protein. Here, both N- and C-terminal coding regions deleted *gppA* clone pGppA18-310 was used for complementation in Δ*gppA* mutant. For the expression of the truncated GppA protein, 0.2% arabinose was used. TLC analysis was done as described in **(B)**. Strains used are Wt, Δ*gppA*, Δ*gppA*(pBADcam), Δ*gppA*(pGppA), and Δ*gppA*(pGppA18-310). The presence of labeled nucleotides including pppGpp and ppGpp is indicated in the left margin.

To determine the importance of the N- and C-terminal regions as well as the conserved domains (I–IV) of the *V. cholerae* GppA protein in pppGpp to ppGpp conversion function, several progressive deletion constructs (Figure 7A) were made by cloning each of the truncated *gppA* ORF under the P*_*BAD*_* promoter of the plasmid pBADcam (Table 1), and each recombinant clone was used for complementation in *V. cholerae* Δ*gppA* strain by assaying the ability of the expressed mutant alleles in intracellular conversion of pppGpp to ppGpp during amino acid starvation. While the Δ*gppA* mutant strains carrying the plasmid pGppA1-313 [Δ*gppA*(pGppA1-313)] or pGppA1-310 [Δ*gppA*(pGppA1-310)], expressing truncated GppA where 184 or 187 amino acids were deleted, respectively, were able to complement the GppA function, the strain Δ*gppA*(pGppA1-272), Δ*gppA*(pGppA1-303), or Δ*gppA*(pBG1-308), where truncated *gppA* expressed 225, 194, or 189 C-terminally amino acids deleted proteins, respectively, failed to complement (Figure 7B). On the other hand, the Δ*gppA* mutant strains carrying the plasmid pGppA13-497 [Δ*gppA*(pGppA13-497)] or pGppA18-497 [Δ*gppA*(pGppA18-497)], expressing N-terminally 12 or 17 amino acids deleted proteins, were able to complement the GppA function but not by the strain Δ*gppA*(pGppA23-497) or Δ*gppA*(pGppA20-497), expressing N-terminally 22 or 19 amino acids deleted GppA, respectively (Figure 7C). Finally, based on the above results, a recombinant plasmid pGppA18-310 (Table 1) containing the truncated *gppA* gene was constructed (Figure 7A), which should express N-terminally (17 amino acids) and C-terminally (187 amino acids) deleted GppA protein. As expected, when the plasmid pGppA18-310 was introduced into the Δ*gppA* mutant, it was able to complement the GppA function (Figure 7D). Thus, it appears that the functional N- and C-terminal boundary of GppA resides between the amino acid positions 18 and 310. In fact, the conserved Ppx-GppA motif actually falls within this minimal functional region of the protein (Figure 2B). To our knowledge this is the first report about functional boundary determination of a bacterial GppA protein.

### Effect of Deletion of the *gppA* Gene on Virulence Phenotypes of *V. cholerae*

We have also examined the effect of deletion of the *gppA* gene on certain virulence phenotypes of *V. cholerae* like cholera toxin production, biofilm formation, and motility. However, no defect in any of these virulence phenotypes was observed in the Δ*gppA* mutant (Supplementary Figure S2).

## Discussion

The *V. cholerae* SR pathway gene *gppA*, the product of which converts pppGpp to ppGpp (Figure 1), has not been characterized previously. Therefore, the main objective of this study was to characterize the *gppA* gene function by genetic and mutational approaches. We found that unlike in *E. coli*, the *gppA* gene in the genome of *V. cholerae* is arranged in an operon along with *rhlB* (Figure 3). In *E. coli*, the *rhlB* gene codes for an ATP-dependent RNA helicase, which is a component of the RNA degradasome complex (Khemici et al., 2005). Although at present no information is available about the function of RhlB of *V. cholerae*, the arrangement of the *rhlB*-*gppA* genes in an operon has raised a possibility that RhlB could be involved in the regulation of the SR in this pathogen, which needs investigation.

We found that the accumulation of pppGpp in Δ*gppA* cells is high (∼10-fold) compared to the isogenic Wt strain during amino acid starvation but not under glucose deficient condition (Figures 4, 5). We have shown earlier that under amino acid starvation, *V. cholerae* RelA synthesizes (p)ppGpp (Haralalka et al., 2003), while during glucose starvation, SpoT and RelV enzymes are responsible for (p)ppGpp synthesis (Das and Bhadra, 2008; Das et al., 2009; Dasgupta et al., 2014). Therefore, it may be argued here that Wt cells produce both pppGpp and ppGpp using GTP and GDP as substrates, respectively, during amino acid starvation, but GppA most likely converts most of the pppGpp molecules to ppGpp (Figure 4). In contrast, under glucose starvation, there was no difference in pppGpp/ppGpp accumulation in Wt and Δ*gppA* cells suggesting that the result is independent of GppA. It may be possible that the affinity of GppA to the pppGpp substrate is altered under glucose starvation stress, or may be some unknown factor is needed to activate the GppA enzyme under this condition, which needs further work. In this context, it may be noted that in *E. coli*, significant amounts of pppGpp accumulate in Δ*gppA* mutant compared to an isogenic Wt strain under both amino acid and glucose starvation conditions (Somerville and Ahmed, 1979). However, our result on glucose starvation of *V. cholerae* cells does not support this finding in *E. coli*, which could be organism specific.

In this study, we have also characterized the *ppx* gene of *V*. *cholerae* to determine whether it has any *gppA*-like function. This is because both Ppx and GppA contain conserved Ppx-GppA motif, which appears to be needed for their similar enzymatic functions (Kristensen et al., 2008). Recently, it has been reported that deletion of the *ppx1* gene of the enteropathogen *Campylobacter jejuni* leads to increase in intracellular accumulation of pppGpp as well as polyP (Malde et al., 2014). We found that the overexpression of Ppx can complement the GppA function in amino acid starved Δ*gppA V. cholerae* cells (Figure 4). Similarly, Kuroda et al. (1997) have reported complementation of the GppA function in an *E. coli* Δ*gppA*Δ*ppkx* mutant strain (GppA-Ppk_Ppx-) by overexpressing the Ppx enzyme. Very recently, the structure of the Ppx/GppA protein of the Gram-negative bacterium *Helicobacter pylori* has been solved (Song et al., 2019). It was found that the *H. pylori* Ppx/GppA enzyme is ∼27-fold less efficient in hydrolyzing pppGpp compared to its polyP hydrolase activity. Analysis of the crystal structures of Ppx and GppA also supports that the active site of Ppx can efficiently bind the narrower polyP but not the wider pppGpp molecule. Similarly, the structure of the active site of GppA allows pppGpp to bind efficiently and hydrolyze it to ppGpp (Song et al., 2019). Thus, it could be plausible that overexpression of *V. cholerae* Ppx in Δ*gppA* cells may hydrolyze pppGpp and complement the *gppA* mutant phenotype due its probable weak pppGppase activity. However, further studies are needed to understand the exact role of Ppx in the complex metabolic network of (p)ppGpp in *V. cholerae.*

Currently, the expression profile of the *gppA* gene in different growth phases is not available for any Gram-negative bacteria. Such analysis indicated that in *V. cholerae*, the *gppA* gene expression is constitutive, although there is modest increase in transcripts level during the stationary growth phase (Figure 6A). This is likely since during stationary phase, bacterial cells face exhaustion of nutrients, which may trigger SR-related gene expression including *gppA*. Since *gppA* expression during amino acid starvation has been observed to be high compared to that of glucose-starved cells (Figure 6B), which could be one of the reasons for no accumulation of pppGpp in amino-acid-starved Wt cells.

After establishing the function of the *gppA* gene, we determined the core functional region of the GppA protein of *V. cholerae* by progressive deletion analysis of the N- and C-terminal regions carrying the conserved domains (I–IV) and Ppx-GppA motif (Figure 2B). However, extensive complementation analysis of N- or/and C-terminal coding deletion alleles allowed us to validate that the Ppx-GppA motif of *V. cholerae* GppA actually spans from amino acid positions 18 to 310 residues, and this is the first report where we have determined the potential minimal functional boundaries of the GppA protein (Figure 7). It is important to mention here that the GppA protein of *A. aeolicus*, the crystal structure of which has been reported, is only 312 amino acids long. On the other hand, in Gram-negative bacteria including *V. cholerae*, GppA is composed of about 500 amino acids (Figure 2B). Our deletion analysis indicated that the Ppx-GppA motif containing the core functional region of *V. cholerae* GppA is 293 amino acids long, which is very close to that of *A. aeolicus* GppA complete protein. As mentioned, the presence of four distinct domains (I–IV) in *E. coli* Ppx and GppA have been reported (Rangarajan et al., 2006; Song et al., 2019). While domains I and II form the active sites of the Ppx and GppA proteins, domain III has similarity with the hydrolase domain of the SpoT enzyme and is probably responsible for the dimer formation of Ppx in *E. coli* (Rangarajan et al., 2006). On the other hand, domain IV showed structural similarities with the cold shock-associated RNA-binding protein (Rangarajan et al., 2006; Song et al., 2019). Our deletion analysis highlights particularly the importance of the conserved N-terminal domains I and II of the GppA protein of *V. cholerae* because disruption of these domains but not the C-terminal domains III and IV leads to functional loss of GppA (Figure 7). Thus, it seems that similar functions are being carried out by the domains I and II of *V. cholerae* GppA protein.

In conclusion, the present study shows that the deletion of *V. cholerae gppA* gene leads to accumulation of substantial amounts of pppGpp molecules during amino-acid-starved condition. At present, however, it is not clearly known which of the alarmones, ppGpp or pppGpp, is most potent in regulating the different phenotypes of SR in bacteria including *V. cholerae*. Since ppGpp is synthesized directly from GDP and almost in all bacteria, the level of intracellular ppGpp under nutritional stress conditions is higher than pppGpp, it is likely that ppGpp is the key player of SR in bacteria (Cashel et al., 1996; Potrykus and Cashel, 2008; Sajish et al., 2009; Mechold et al., 2013). In addition, the presence of functional GppA in Gram-negative bacteria further supports the hypothesis that ppGpp is the critical molecule for controlling SR in bacteria. It also appears from this study that the activation of the RelA and GppA enzymes are probably linked with amino acid starvation condition, which may help in maintaining the lower pppGpp level in *V. cholerae*. Further studies on other Gram-negative bacteria may help in establishing this unique link between RelA and GppA.

## Data Availability Statement

Data presented in this study are included in the article/Supplementary Material, further inquiries can be directed to the corresponding authors.

## Author Contributions

RKB and BD designed the study. DR and SD performed the experiments. DR, SD, BD, and RKB analyzed the data. DR, BD, and RKB wrote the manuscript. All authors have contributed to the article and approved the submitted version.

## Conflict of Interest

The authors declare that the research was conducted in the absence of any commercial or financial relationships that could be construed as a potential conflict of interest.

## Footnotes

1 https://imagej.nih.gov

2 http://www.technelysium.com.au/chromas.html

3 www.jcvi.org

4 www.genome.jp/kegg/

5 www.ncbi.nlm.nih.gov

6 https://www.ebi.ac.uk/Tools/msa/clustalo/

7 www.psc.edu/biomed/genedoc

8 http://frodo.wi.mit.edu/

9 https://blast.ncbi.nlm.nih.gov/blast.cgi

10 http://biocyc.org/VCHO/
