# Supplementary material for: Functional Insights Into the Role of gppA in (p)ppGpp Metabolism of Vibrio cholerae
Source: Front Microbiol. 2020 Sep 29;11:564644. doi: 10.3389/fmicb.2020.564644 (PMC7552824; doi:10.3389/fmicb.2020.564644)
Supplement: Supplementary file 1 [file Presentation_1.pdf]

Supplementary Information

to

Functional Insights into the Role of *gppA* in (p)ppGpp Metabolism of *Vibrio cholerae*

Dipayan Rakshit[1], Shreya Dasgupta[1], Bhabatosh Das[2]* and Rupak K. Bhadra[1]*

[1]*Infectious Diseases and Immunology Division, CSIR-Indian Institute of Chemical Biology, Kolkata, West Bengal, India*

[2]*Molecular Genetics Laboratory, Infection and Immunology Division, Translational Health Science and Technology Institute, Faridabad, Haryana, India*

E-mails: rupakbhadra@iicb.res.in; bhabatosh@thsti.res.in

Running Title: *V. cholerae* GppA and (p)ppGpp metabolism

Materials and Methods

Growth Assay

For comparison of the growth of *V. cholerae* Δ*gppA* mutant with the Wt strain N16961 in nutrient rich versus nutrient poor medium, bacterial cells were grown in LB and in M9 minimal medium (Sigma-Aldrich, United States) containing 0.4% glucose as a carbon source at 37°C with shaking essentially as described previously (Das and Bhadra, 2008). Growth of bacterial culture was monitored at 1 h interval by measuring the optical density (OD) at 600 nm (OD_{600}) using a spectrophotometer (Hitachi, Model U-5100, Japan) and the OD values of Δ*gppA* and Wt strains were plotted against time. Each experiment was repeated thrice and average values were plotted.

Motility Assay

Motility assay for *V. cholerae* strains was carried out on LB soft-agar plates containing 0.3% agar (Difco, United States) at 30°C as described previously (Pal et al., 2012). Reading was taken after 5 h of incubation. Experiments were repeated thrice and the average values were plotted.

GM_1-ELISA Assay for the Detection of Cholera Toxin (CT)

For the detection of CT production, *V. cholerae* strains were grown in AKI medium at 37°C for 8 h with shaking essentially as described previously (Pal et al., 2012; Basu and Bhadra, 2019). CT present in the culture supernatant was assayed by GM_1-enzyme linked immunosorbent assay (ELISA) using pure CT (Sigma-Aldrich, United States) and phosphate-buffered saline (10 mM, pH 7.2) as positive and negative controls, respectively. A standard curve of known CT concentrations was plotted and used to estimate the amount of CT present in each sample.

Biofilm Assay

Biofilm assay was performed as described previously (Pratt et al., 2007) with some modification. In brief, *V. cholerae* cells were grown in LB using a 96-well polystyrene microtiter plate at 30°C for 24 h without shaking. The medium was aspirated and adherent biofilm was gently washed three times with distilled water. Biofilm was stained with 1 mg mL^{-1} crystal violet (Sigma-Aldrich, United States) for 10 min, washed extensively with distilled water. Biofilm bound

crystal violet was solubilized with dimethylsulphoxide (Sigma-Aldrich, United States) and quantified by absorbance at 570 nm using a microtiter plate reader (Model Multiskan EX, Thermo Scientific, United States). Each data point was averaged of replicate wells from three independent experiments and represented as OD_{570}/OD_{600} ratio so as to nullify growth defect of the cells if any (He et al., 2012).

References

Basu, P., and Bhadra, R. K. (2019). Post-transcriptional regulation of cholera toxin production in *Vibrio cholerae* by the stringent response regulator DksA. *Microbiology*. 165, 102-112.

Das, B.,and Bhadra, R. K. (2008). Molecular characterization of *Vibriocholerae* Δ*relA* Δ*spoT* double mutants. *Arch. Microbiol*.189, 227-238.

He, H., Cooper, J. N., Mishra, A., and Raskin, D. M. (2012). Stringent response regulation of biofilm formation in *Vibrio cholerae*. *J. Bacteriol.* 194, 2962-2972.

Pal, R. R., Bag, S., Dasgupta, S., Das, B., and Bhadra, R. K. (2012). Functional characterization of the stringent response regulatory gene *dksA* of *Vibrio cholerae* and its role in modulation of virulence phenotypes. *J. Bacteriol*. 194, 5638-5648.

Pratt, J. T., Tamayo, R., Tischler, A. D., and Camilli, A. (2007). PilZ domain proteins bind cyclic diguanylate and regulate diverse processes in *Vibrio cholerae*. *J. Biol. Chem.* 2282, 12860-12870.

Table S1. Primers used in this study.

Primer Name	Sequence (5'-3')	Reference
ppxMut-F1	CCGGAGCTCTTCATATGGTGGTCGCC<br>*Sac*I	This study
ppxMut-R1	AACTGCAGATGATCATTTCCGTG<br>*Pst*I	This study
ppxMut-F2	CCCAAGCTTGAATTCTTGGCGCAAG<br>*Hin*dIII	This study
ppxMut-R2	GGGGTACCGACTTCACGCCAGTATTC<br>*Kpn*I	This study
gppAorf- F	CGTGAATTCGCTATGAGCCAAGCCGTTTC<br>*Eco*RI	This study
gppAorf-R	AACTGCAGCTATACGCTGCCTACTTGC<br>*Pst*I	This study
ppxORF-F	GCTCTAGATTGCCATTTACGACTATC<br>*Xba*I	This study
ppxORF-R	CCCAAGCTTATTGCAGTGACTAGTCTG<br>*Hin*dIII	This study
gppAmut-F1	GGGGTACCTATTGGCCGTACGGGTCG<br>*Kpn*I	This study
gppAmut-R1	CGGAATTCTGTTGGAACCTAAATC<br>*Eco*RI	This study
gppAmut-F2	CGGAATTCGTACGTCGCTATCGCG<br>*Eco*RI	This study
gppAmut-R2	CCGGAGCTCAGATGAGGGCTTAATAC<br>*Sac*I	This study
gppA rtm-F	GCAAAGAGGGTGGGATTGTC	This study
gppA rtm-R	TCGGATATTCTCTGCGGGAAT	This study
gppA-R	CAGCATGGCTTGCTTTTGTA	This study
rhlB-F	ATCGCATGTTTGATCTCGGC	This study
rhlB-R	ACATCACCAGTCAGTAGGCC	This study
gppA Seq-F	TCTCGCAATAGCAATAATG	This study
GppA272-R	AACTGCAGTTAAATCGATAGGCCACTTGG<br>*Pst*I	This study
GppA303-R	AACTGCAGTTATTGAACCATCTCATAGAC<br>*Pst*I	This study
GppA308-R	AACTGCAGTTATTCTTGGCGTAGGTCTTG<br>*Pst*I	This study
GppA310-R	AACTGCAGTTAAATATCTTCTTGGCGTAG<br>*Pst*I	This study
GppA313-R	AACTGCAGTTAGCGCGCTCGAATATCTTC<br>*Pst*I	This study
GppA13-F	CGGAATTCGCCATGATTGATTTAGGTTCCAAC<br>*Eco*RI	This study
GppA18-F	CGGAATTCTCCATGAACAGTTTTCACATGTTG<br>*Eco*RI	This study
GppA20-F	CGGAATTCAGTATGTTTCACATGTTGGTCGTG<br>*Eco*RI	This study
GppA23-F	CGGAATTCCACATGTTGGTCGTGCGTCATATC<br>*Eco*RI	This study
recA-F	GCAATTTGGTAAAGGCTCCA	Pal et al., 2012
recA-R	GTTGTGCAGCAGCAATCAGT	Pal et al., 2012

gyrArtm-F	AACAACCTGTATGCCAACACTCA	This study
gyrArtm-R	TCGAGTGCCACCATGTTGAT	This study
vpsRrtm-F	GCTGGATGAGTCTCAGCTCGAT	This study
vpsRrtm-R	CGTTCCCGAATGCTTTTCA	This study
vpsTrtm-F	CTCCATTATCGTGCCGGTAAC	This study
vpsTrtm-R	CTTGATAATCTGTTGTTCTCTTTTGGTT	This study

Restriction enzyme sites are underlined.

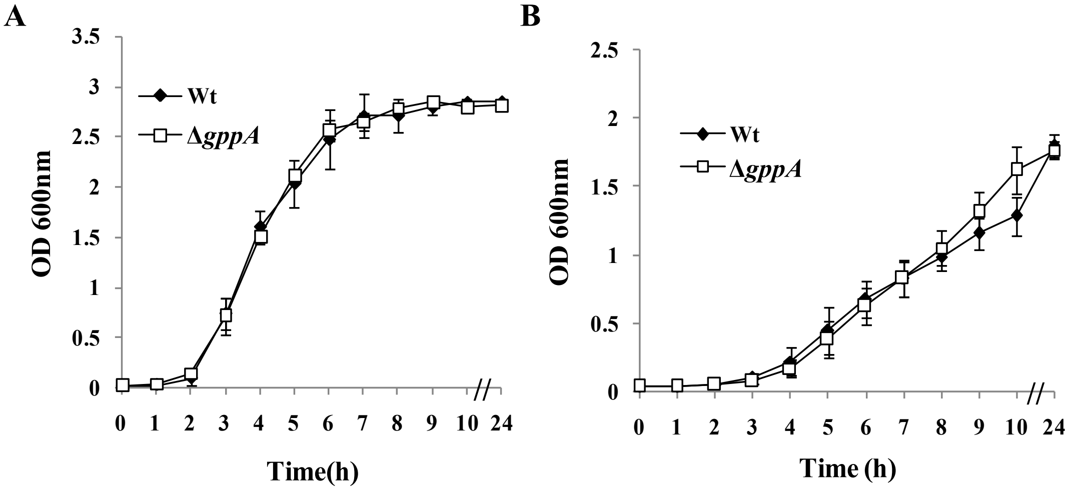

Figure S1. Growth phenotypes of Δ*gppA* strain of *V. cholerae*. Growth of *V. cholerae* strains in **(A)** nutrient rich LB medium, **(B)** M9 minimal medium. In each experiment Wt *V. cholerae* strain N16961was used as a control. Each experiment was repeated thrice and average values were plotted. Error bar indicates standard deviations.

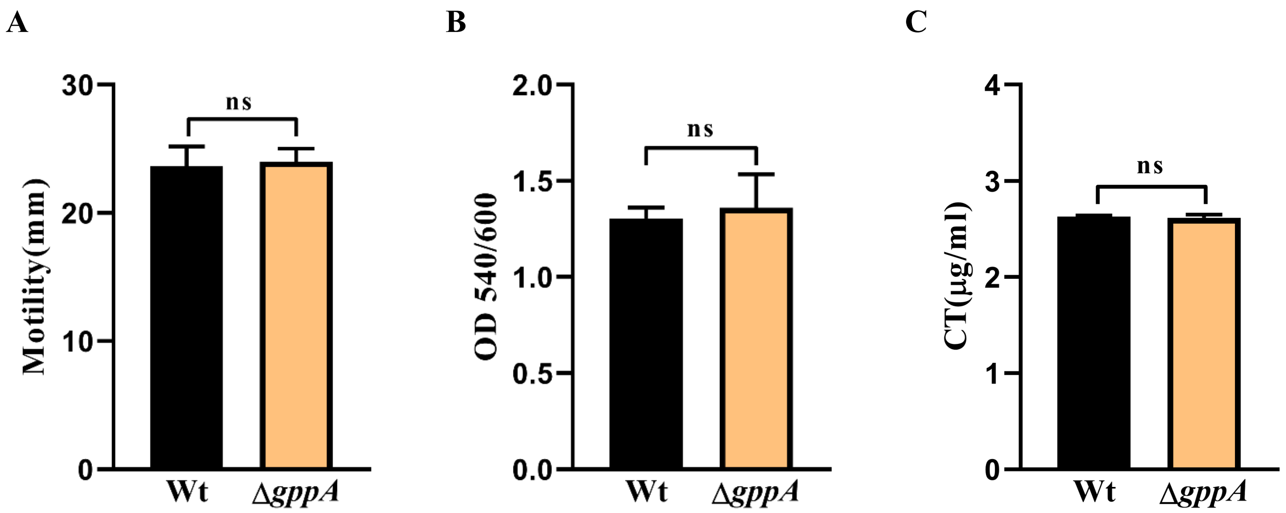

Figure S2. Mutation of the *gppA* gene of *V. cholerae* has no effect on the motility, biofilm formation and CT production. **(A)** motility**, (B)** biofilm formation and **(C)** CT production by *V. cholerae* Δ*gppA* mutant as indicated. In each experiment Wt *V. cholerae* strain N16961was used as a control. Experiments were repeated thrice and the average values were plotted. Error bars indicate standard deviations. (ns = non-significant).
